# A Combination of Schwann-Cell Grafts and Aerobic Exercise Enhances Sciatic Nerve Regeneration

**DOI:** 10.1371/journal.pone.0110090

**Published:** 2014-10-15

**Authors:** Camila Oliveira Goulart, Sofia Jürgensen, Allana Souto, Júlia Teixeira Oliveira, Silmara de Lima, Chiara Tonda-Turo, Suelen Adriani Marques, Fernanda Martins de Almeida, Ana Maria Blanco Martinez

**Affiliations:** 1 Laboratório de Neurodegeneração e Reparo, Departamento de Patologia, Faculdade de Medicina, HUCFF, UFRJ, Rio de Janeiro, RJ, Brazil; 2 Department of Mechanics, Politecnico di Torino, Turin, Italy; 3 Instituto de Biologia, Departamento de Neurobiologia, Laboratório de Regeneração Neural e Função, UFF, Rio de Janeiro, Brazil; 4 Pólo Universitário Macaé, UFRJ, Macaé, RJ, Brazil; Federal University of Rio de Janeiro, Brazil

## Abstract

**Background:**

Despite the regenerative potential of the peripheral nervous system, severe nerve lesions lead to loss of target-organ innervation, making complete functional recovery a challenge. Few studies have given attention to combining different approaches in order to accelerate the regenerative process.

**Objective:**

Test the effectiveness of combining Schwann-cells transplantation into a biodegradable conduit, with treadmill training as a therapeutic strategy to improve the outcome of repair after mouse nerve injury.

**Methods:**

Sciatic nerve transection was performed in adult C57BL/6 mice; the proximal and distal stumps of the nerve were sutured into the conduit. Four groups were analyzed: acellular grafts (DMEM group), Schwann cell grafts (3×10^5^/2 µL; SC group), treadmill training (TMT group), and treadmill training and Schwann cell grafts (TMT + SC group). Locomotor function was assessed weekly by Sciatic Function Index and Global Mobility Test. Animals were anesthetized after eight weeks and dissected for morphological analysis.

**Results:**

Combined therapies improved nerve regeneration, and increased the number of myelinated fibers and myelin area compared to the DMEM group. Motor recovery was accelerated in the TMT + SC group, which showed significantly better values in sciatic function index and in global mobility test than in the other groups. The TMT + SC group showed increased levels of trophic-factor expression compared to DMEM, contributing to the better functional outcome observed in the former group. The number of neurons in L4 segments was significantly higher in the SC and TMT + SC groups when compared to DMEM group. Counts of dorsal root ganglion sensory neurons revealed that TMT group had a significant increased number of neurons compared to DMEM group, while the SC and TMT + SC groups had a slight but not significant increase in the total number of motor neurons.

**Conclusion:**

These data provide evidence that this combination of therapeutic strategies can significantly improve functional and morphological recovery after sciatic injury.

## Introduction

Injuries to the peripheral nervous system affect millions of people around the world and reduce their motor skills. Although the peripheral nervous system has some regenerative potential, complete functional recovery is seldom achieved, especially after severe lesions that lead to interruption of nerve continuity and tissue loss [1]. In these cases, the most common therapeutic approach is the use of an autologous nerve graft; however, this technique has a number of disadvantages, such as donor site morbidity and limited functional recovery [2], [3].

Different potential strategies to stimulate peripheral axonal growth have been proposed, among them is the use of cell therapy, which has been deemed efficient mainly due to the release of trophic factors [4]–[6]. Exogenous Schwann cells have the potential to aid regeneration of nerve fibers, not only due to its key role in myelination, but also for their ability to secrete trophic factors that promote survival and axonal growth [7]–[11]. Additionally, the use of a biodegradable conduit to guide axonal growth, insulate and protect the injury site from negative influences is also a promising alternative to nerve grafts. It has been well documented that this type of conduit represents a suitable substrate for survival and differentiation of Schwann cells. In addition, its association with different cell types such as fibroblasts, bone marrow-derived cells or Schwann cells can maintain trophic factors at the lesion site and can help to improve outcomes in peripheral nervous system repair [12]–[14]. Another potent therapeutic strategy is treadmill training, which has been shown to improve motor function after spinal cord injury both in animals and human subjects [15]–[17]. The effects of treadmill training on functional recovery following peripheral nerve injuries were described recently [18], [19]. Using different training paradigms, some authors have found that treadmill training promotes functional recovery, due to an increase in release of trophic factors and the stimulation of axonal growth [20], [21].

The faster regeneration occurs, the more likely it is that the target organ will be reinnervated, as the axonal growth rate tends to decrease over time [22]. To date, few studies have given attention to combining different approaches in order to accelerate the regenerative process. The present study has tested the effectiveness of combining Schwann-cell transplantation into a biodegradable conduit, with treadmill training as a therapeutic strategy to improve the outcome of repair after mouse sciatic nerve transection.

Our findings suggest that this combination of therapeutic strategies can significantly improve functional and morphological recovery. Moreover, we show that transplanted Schwann cells can be functionally incorporated into the regenerated tissue and have the ability to secrete neurotrophic factors. These factors, either induced by physical exercise or directly released by transplanted Schwann cells, are increased by the combination of therapies, most likely underlying the benefit of the association.

## Methods

### Animals

Thirty-two male C57Bl/6 mice weighing 20–25 g were randomly divided into four groups: those with acellular grafts (n = 8, culture medium Dulbeco's Modified Eagle Medium, DMEM group), those with Schwann cell grafts (n = 8, SC group), those with treadmill training (n = 8, TMT group), and those with treadmill training and Schwann cell grafts (n = 8, SC + TMT group). Animals were subjected to sciatic nerve transection and tubulization with either a polycaprolactone (PCL) or collagen conduit. All animal use and care protocols were approved by the Ethics Committee for the Use of Experimental Animals of the Center of Health Sciences of the Federal University of Rio de Janeiro, Brazil (Protocol DHE003).

### Schwann cells: Isolation and purification

Schwann cells were obtained from C57Bl/6 mice expressing green fluorescent protein (GFP + mice), allowing the subsequent identification of these cells, as previously described [23]. Briefly, a 10-mm segment of sciatic nerves and brachial plexus of GFP+ mice were removed and dissected, the epineurium was stripped off, and nerve segments were allowed to degenerate *in vitro*. After 3 days, degenerated segments were enzymatically and mechanically dissociated, washed, and re-suspended in culture medium containing 20 µM pituitary extract (Invitrogen, USA) and 1 µM forskolin (Sigma); dissociated cells were seeded on plates pre-coated with laminin (10 µg/mL for 1 h) (Sigma). After 24 h, the medium was changed to remove cellular debris, and 100 µM AraC (Sigma) was added for another 24 h to prevent fibroblast proliferation. The medium was changed twice a week until optimal Schwann cell confluence was reached. Three expansion cycles were performed before cells were harvested, counted and injected into the conduit, at a density of 3×10^5^ cells in 2 µL of DMEM-F12.

### Surgical procedure

Surgeries were performed under deep anesthesia using i.p. injections of ketamine (100 mg/kg) and xylazine (15 mg/kg). The left sciatic nerve was exposed and transected at mid-thigh level. First, 1 mm of the proximal stump was sutured into a polycaprolactone or collagen conduit (5 mm long), through the perineurium, using a 10.0 monofilament nylon suture. Subsequently, the graft was performed in the conduit for each group as follows: groups SC + TMT and SC the conduit was grafted with Schwann cells in DMEM (3×10^5^/2 µL), and the TMT and DMEM groups received only DMEM (2 µL). Grafts were injected into the conduit using a 10-µL Hamilton microsyringe. Finally, the distal stump was sutured through the perineurium to the other end of the conduit, leaving a 3-mm gap between the stumps. Polycaprolactone conduits were provided by Dr. Chiara Tonda-Turo, from Politecnico di Torino, Turin, Italy [24]. Collagen conduits were previously used and described by our group [25].

Eight weeks later, the animals were anesthetized with ketamine and xylazine (as above) and euthanized by intracardiac perfusion with a fixative solution (4% paraformaldehyde in 0.1 M phosphate buffer, pH 7.4). After perfusion, the regenerated left sciatic nerve within the conduit, the L4 spinal cord, de L4 dorsal root ganglion (DRG) and de gastrocnemius muscles were harvested and processed for immunohistochemistry, light or electron microscopy.

### Treadmill training

Animals were subjected to a one-week familiarization to the treadmill apparatus before experimental protocols began. Treadmill training started on the third day after transection and surgical repair of the sciatic nerve [26], [27]. Mice were placed on a motor-driven treadmill (Insight, Ribeirão Preto, Brazil) at a belt speed of 10 m/min and were trained in two 30-min exercise periods with a 10-min rest period between, 3 days per week, as described previously, with few modifications [28]. Mice ran on the treadmill at this speed with little or no persuasion, even on the third post operative day. Training protocol was conducted during the 8-week survival period. The animals in the DMEM and CS groups were handled and placed on a treadmill with no motion at the same times as the exercise groups.

### Functional assessment

Motor function was evaluated with the sciatic functional index (SFI) as previously described [29]–[32] each week after surgery, the animals' pawprints were recorded, and two measurements were taken: (i) the print length (PL), corresponding to the distance from the heel to the third toe; and (ii) the toe spread (TS), corresponding to the distance from the first to fifth toe. Both measurements were taken from injured (E, for experimental) as well as noninjured (N, for normal) sides, and the SFI was calculated according to the following equation: 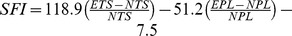


The sciatic function index oscillates around zero for normal nerve function, and when it is equal to −100 represents total loss of function of the nerve.

We also performed the global mobility test (GMT) [33]. The animals were acclimated to the open field prior to injury, and at 2, 4, 6 and 8 weeks after surgery they were submitted to global mobility test. Global mobility was assessed by videotape with a WebCam (5 frames/s, for 1 min; KMEX, USA) using the free K3CCD software, and quantified using ImageJ free software.

### Scanning electron microscopy

The ultrastructural features of the polycaprolactone conduit were investigated before and after 3 and 8 weeks of implant, its proximal portion was fixed by immersion, then washed in 0.1 M phosphate buffer (pH 7.4) and 0.1 M cacodylate buffer (pH 7.4), and postfixed for 2 h in 1% osmium tetroxide containing 0.8% potassium ferrocyanide and 5 nM calcium chloride in 0.1 M cacodylate buffer (pH 7.4). Then, it was washed in 0.1 M cacodylate buffer (pH 7.4), dehydrated in an ethanol series, gold-sputtered (FL-9496 Balzers, Union Coater) and observed in a Jeol JSM-5310 scanning electron microscope.

### Immunoelectron microscopy

The regenerated sciatic nerve was divided into proximal, middle and distal portions. Middle segments of nerve were immersed overnight in fresh fixative solution (4% paraformaldehyde in 0.1 M phosphate buffer pH 7.4). Tissue was washed in phosphate buffer (pH 7.4), dehydrated through a graded ethanol series, embedded in LRWhite resin (London Resin Company) and polymerized at 50°C, for 24 h. Ultrathin sections were obtained on an RMC ultramicrotome and collected on nickel grids. All sections were blocked with a blocking solution (PBS, 1% bovine serum albumin and 0.5% milk) for 40 min and incubated in primary antibodies (anti-GFP, Millipore, 1∶50) for 3 h at room temperature. After rinsing for 20 min in blocking solution, sections were incubated in the secondary antibody (BBI, goat anti-mouse IgG conjugated to 10-ηm gold particles, 1∶100) for 2 h, washed in PBS for 30 min, washed 30 min in distilled water, and stained with uranyl acetate and lead citrate. Primary antibodies were omitted for the negative staining controls. The sections were observed and photographed in a Jeol (JEM 101) transmission electron microscope.

### Light microscopy and transmission electron microscopy

After nerve middle segments were fixed by immersion in 2.5% glutaraldehyde, they were washed in 0.1 M cacodylate buffer (pH 7.4), and postfixed for 90 min in 1% osmium tetroxide containing 0.8% potassium ferrocyanide and 5 mM calcium chloride in 0.1 M cacodylate buffer (pH 7.4). The segments were then washed in 0.1 M cacodylate buffer (pH 7.4) and stained in 1% uranyl acetate overnight. Nerves were dehydrated in graded acetone, infiltrated with Embed-812 resin (Electron Microscopy Sciences) and polymerized at 60°C for 48 h. Semi-thin (500 ηm) and ultra-thin (70 ηm) cross-sections were obtained on an RMC MT-6000 ultramicrotome. The semi-thin sections were stained with 1% Toluidine Blue and examined under a light microscope (Zeiss Axioskop 2 Plus); ultrathin sections were collected on copper grids, contrasted in 5% uranyl acetate and 1% lead citrate, and analyzed in a transmission electron microscope operated at 80 kV (Jeol 900).

### Morphometric assessment

To quantify the total number of myelinated fibers in each regenerated nerve, photographs of the semi-thin cross sections were taken under the light microscope at 40x magnification, using the program Axiovision Rel. 4.5. The entire nerve was photographed, and myelinated fibers were counted with the use of ImageJ Software (1.42q, USA).

Samples of five areas from the same semi-thin cross sections were obtained at a magnification of 100x. For each sample the following parameters were calculated: number of blood vessels, fiber area, axon area, myelin area and G ratio. Myelin area was measured by subtracting the axon area from the fiber area. The G ratio was calculated by dividing the axon diameter by the fiber diameter, and the results were stratified in ranges of 0.0–0.4, 0.4–0.5, 0.5–0.6, 0.6–0.7, 0.7–0.8 and 0.8–0.9. For each range, the lowest value was included and the highest one excluded (e.g., the 0.0–0.4 range includes 0.0 through 0.399, excluding 0.4).

### Immunohistochemistry and imaging

To investigate the integrity of neuromuscular junctions (NMJs), the gastrocnemius muscle was immunolabeled for neurofilaments and counter-stained with anti-α-bungarotoxin. After the tissue was fixed, it was washed in 0.1 M PBS (pH 7.4), cryoprotected in 10%, 20%, 30% PBS-sucrose, embedded in OCT (Tissue-Tek, Tokyo, Japan) and frozen. Sixty-µm longitudinal frozen sections were made on a cryostat (Leica CM 1850, Germany), collected and washed in PBS, treated with ammonium chloride solution (NH4Cl) for 1 h, washed in PBS for 15 min, incubated in blocking solution (10% normal goat serum, 3% bovine serum albumin, in PBS 0.1 M plus 0.3% Triton) for 1 h, and washed in PBS-Triton. Slides were incubated in the primary antibodies rabbit anti-NF200 (Sigma, 1∶200) and anti-α-Bungarotoxin (conjugated with Alexa Fluor 594, Molecular Probes, 1∶500), overnight at 4°C in a humid chamber. Slides were washed in PBS for 30 min, incubated in the secondary antibody (Alexa Fluor 488 goat anti-rabbit, Sigma, 1∶600) for 2 h at room temperature, and washed in PBS for 30 min. Finally, the slides were mounted with Fluoromount (Sigma).

The sciatic nerve distal segments, the L4 dorsal root ganglion and L4 spinal cord were processed following the same protocol for cryopreservation described above. Frozen longitudinal serial sections of dorsal root ganglions (12-µm) and transverse serial sections of sciatic nerve (10-µm) and the spinal cord (20-µm) were made and collected on gelatin-coated glass slides. To investigate the presence of Schwann cells in the host tissue, slides were washed in PBS, incubated in blocking solution, and then in the rabbit anti-S-100 primary antibody (Sigma, 1∶200). Sections were washed and incubated in the secondary antibody (Alexa 488 goat anti-rabbit, Sigma, 1∶600) for 2 h. Nuclei were stained with DAPI (Molecular Probes, USA, 1∶10,000) and mounted with Fluoromount (Sigma).

To analyze the levels of Brain-Derived Neurotrophic Factor (BDNF), Nerve Growth Factor (NGF), and Neurotrophins 3 and 4 (NT-3, NT-4) present both *in vitro* and *in vivo*, we used the following primary antibodies: rabbit anti-human BDNF (Preprotech, 1∶100), rabbit anti-human NGF (Preprotech, 1∶100), goat anti-mouse NT-3 (Preprotech, 1∶100) and goat anti-mouse NT-4 (Preprotech, 1∶100). After incubation in primary antibodies, sections were washed and incubated in secondary antibodies: Alexa 488 goat anti-rabbit (Sigma, 1∶600) or Alexa 488 rabbit anti-goat. Slides were washed and coverslipped with Fluoromount.

Primary antibodies were omitted for all negative staining controls. Staining was analyzed in a Zeiss Axioskop 2 Plus fluorescence microscope, equipped with a Zeiss Axiocam MRC camera, and Axiovision version 4.5 was used for image acquisition. For trophic factors immunostaining analysis, a spinning disk confocal microscope (Leika TCS SP5 AOBS) was used.

### Trophic factor quantification

To quantify tissue levels of BDNF, NGF, NT-3 and NT-4, images were collected from all immunostained sections using a 20x objective, and analyzed using the Image-Pro Plus software (Media Cybernetics, version 6.0). After proper threshold setting, quantification was done by measuring the ratio between the stained area and the total field area [34]. A total of eight sections per nerve were analyzed, from five animals from each group.

### Neuronal quantification

For quantification of sensory and motor neurons, slides with dorsal root ganglion and spinal cord (6–8 sections per slide) were stained with cresyl violet. Sections were photographed on a Zeiss Axioskop 2 plus microscope at a 20x magnification. All neurons nucleoli in the DRGs sections and in the ventral horn of the spinal cord were counted using ImajeJ Software (1.42q). To avoid double counting of neurons, the distance between the sections used for dorsal root ganglion and L4 spinal cord segment quantification was 96 and 200-µm, respectively. To ensure that only motor neurons were counted, a perpendicular line was drawn in the spine central axis, immediately in front of the spinal cord central channel.

### Statistical analysis

All analyses were carried out using GraphPad Prism 5.01 (GraphPad Software, Inc.). Statistical analyses used One-Way analysis of variance for comparisons between four groups, followed by the Tukey post-hoc test when necessary. When the values did not assume Gaussian distribution or had large coefficients of variation, the chosen test was Kruskal-Wallis nonparametric test, followed by Dunn's post-hoc test. Two-Way analysis of variance was used for comparisons between four groups during the survival time. Data are presented as mean ± standard error of the mean (SEM). A *P*-value <0.05 was considered significant.

## Results

### Combined therapies improve nerve regeneration both functionally and morphologically

**Figure 1:**
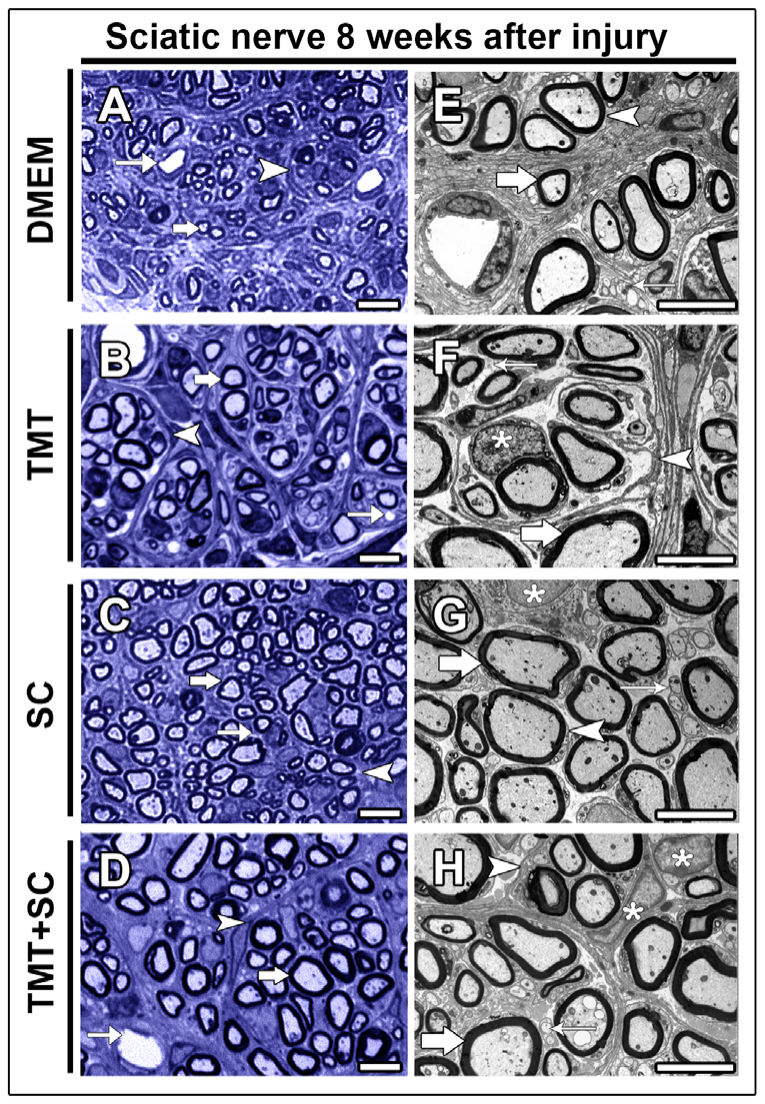
Combined therapies improve nerve regeneration. (A–D) Semi-thin cross sections of regenerating sciatic nerves. (A) DMEM group with clusters of regenerating nerve fibers (arrowhead), myelinated fibers (large arrow) and blood vessels (thin arrow). (B, C and D) TMT, SC and TMT + SC-treated groups display nerves with many clusters of regenerating fibers (arrowheads) with myelinated nerve fibers (thick arrows) and blood vessels (thin arrows). Scale bar  = 20 µm. (E–H) Transmission electron micrographs of regenerating nerves. (E) DMEM group; cross section showing small and poorly developed regenerating clusters (arrowhead) composed of thin, dispersed myelinated nerve fibers (thick arrow) and non-myelinated nerve fibers (thin arrow). (F, G and H) TMT, SC and TMT + SC-treated groups showing regenerating clusters consisting of myelinated (thick arrows) and non-myelinated nerve fibers (thin arrows) surrounded by processes of perineurium-like cells (arrowheads). Schwann-cell nuclei are also observed (asterisks). Scale bar  = 2 µm.

**Figure 2:**
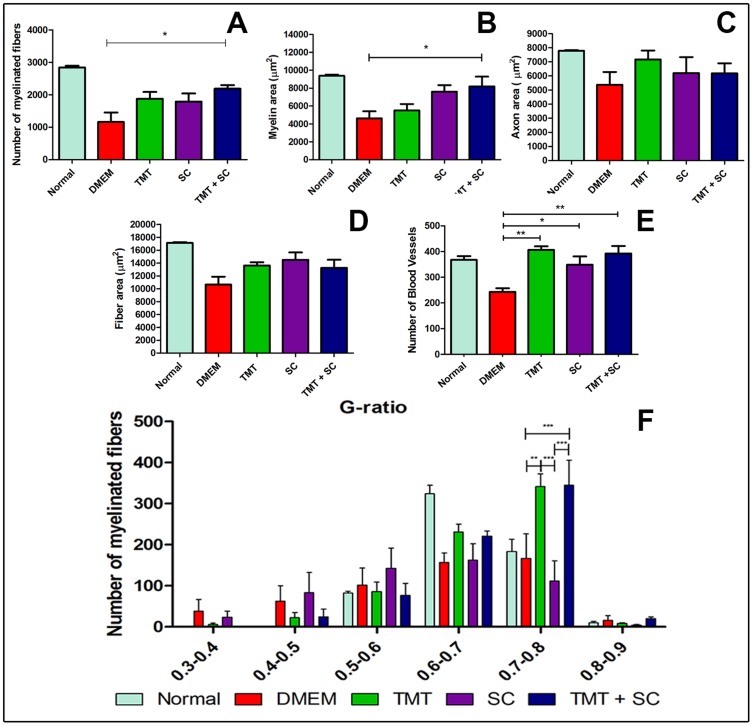
Quantitative analyses of the number of myelinated nerve fibers, myelin area, axon area and fiber area. (A–D; *P<0.05) (E) Quantitative analysis of the number of blood vessels. (F) G-ratio analyses stratified by ranges. Values represent mean ± SEM, *P<0.05, **P<0.01 and ***P<0.001.

Semi-thin sections of the regenerating sciatic nerves revealed the best overall organization in the group that received the combined therapies, compared with other groups (Figure 1A–D). The ultrastructural analysis (Figure 1E–H) confirmed the light microscopy observation. Quantification of morphological parameters (Figure 2A–F) showed that the number of myelinated nerve fibers (Figure 2A) and myelin area (Figure 2B) differed significantly in the TMT + SC group (2060±92.10 and 8195±1090, respectively; *P*<0.05) compared to the DMEM group (1324±279.9 and 4628±781, respectively; *P*<0.05). However, the analyses of the axon and fiber areas (Figure 2C and 2D, respectively) failed to show a significant difference between the groups. The number of blood vessels was significantly increased in the TMT, SC and TMT + SC groups (Figure 2E; 406.7±13.53, 348.4±32.51 and 392.4±28.9, respectively, *P*<0.05; *P*<0.01) compared to DMEM group (243±14.33). G-ratio analysis showed that DMEM and SC groups displayed many fibers in the range of 0.4 to 0.6, whereas TMT and TMT + SC groups achieved the best results, with most fibers in the range 0.5 to 0.8, the most suitable G-ratio index for the sciatic nerve (Figure 2F).

**Figure 3:**
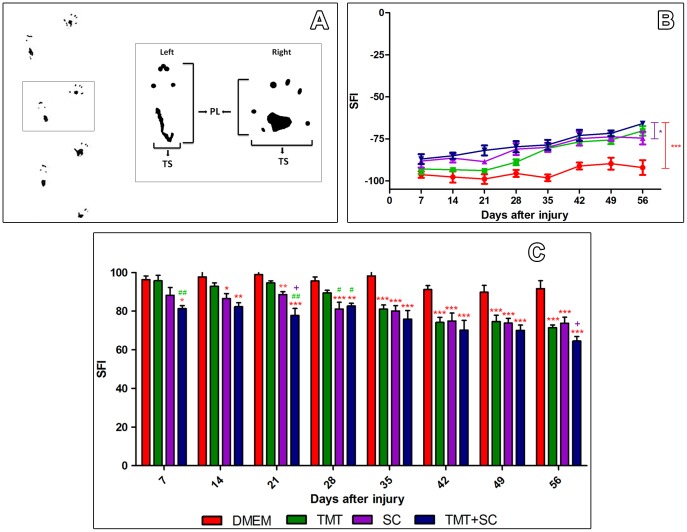
Functional recovery is improved with TMT + SC combined therapy. (A) Measurements of print length (PL) and toe spread (TS), exemplifying the values used to measure the footprint. (B) Line graph showing the variation of the sciatic functional index (SFI) over time. (C) Bar graph (normalized to positive values), showing statistically significant differences between the treated groups and DMEM (represented by *); between the TMT and TMT + SC groups (represented by #), and between the TMT + SC and SC groups (represented by +). The TMT + SC group had a better functional outcome, indicated by the significant difference at 7 days after injury.

**Figure 4:**
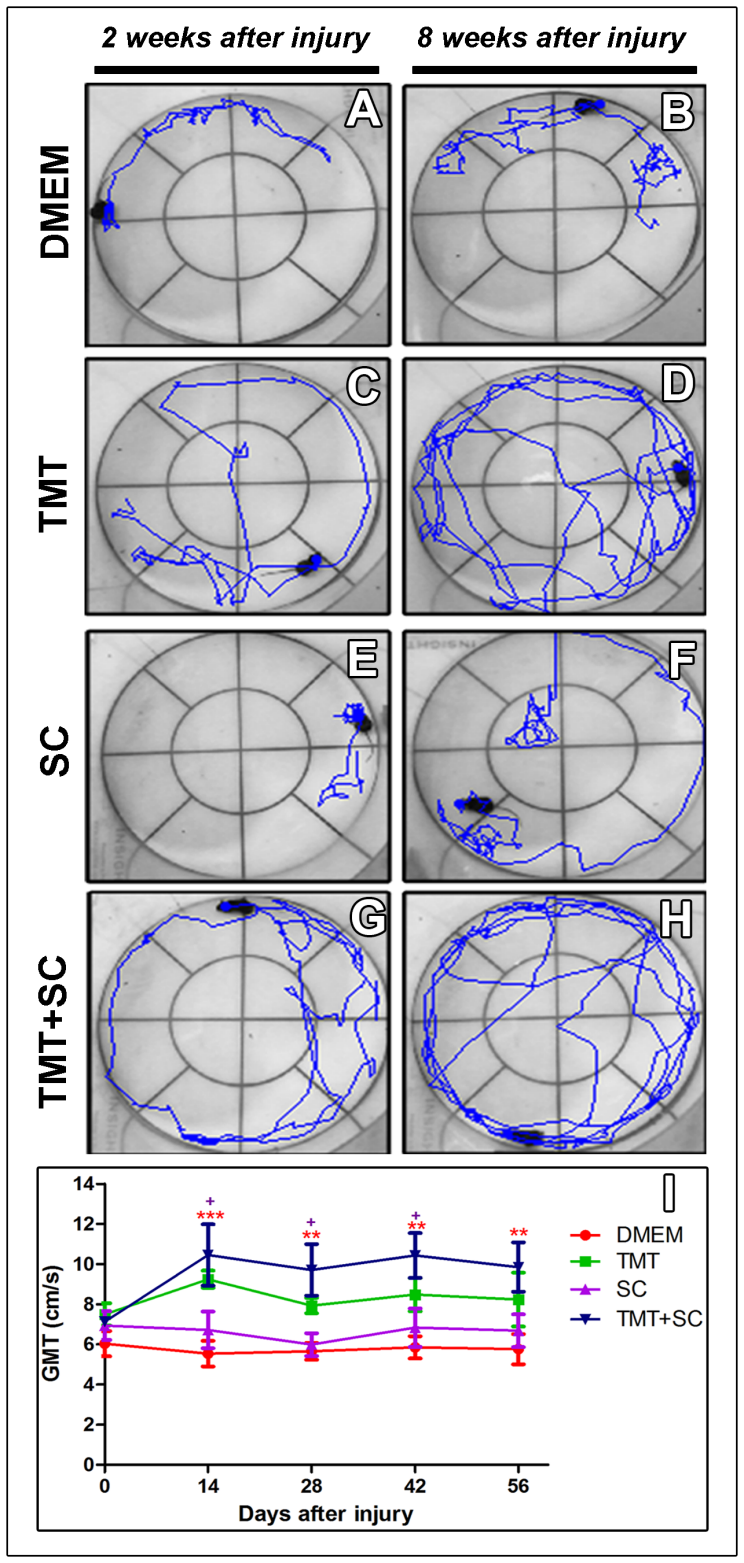
Functional analysis of global mobility test (GMT). (A and B) DMEM group; (C and D) TMT group; (E and F) SC group; (G and H) TMT + SC group; at different times after surgery (14 and 56 days). In general, treated groups showed improved exploratory capacity through the open field. (I) GMT analysis indicated that TMT and TMT + SC-treated animals were able to move faster compared to the DMEM and SC-treated animals. Values represent mean ± SEM, *P<0.05, **P<0.01, ***P<0.001.

To assess the efficacy of therapies on motor function, we used the sciatic function index and the global mobility test (Figures 3 and 4). Although all animals showed broad loss of function in the first week after injury, the TMT + SC group showed significant improvement compared to the DMEM and TMT groups (Figure 3B and 3C). Thereafter, all groups showed progressive improvement in motor function, but TMT, SC and TMT + SC groups showed higher values of SFI during the entire evaluation period (Figure 3B and 3C). In the last week, all treatments resulted in a statistically significant functional improvement compared to DMEM group. Data from global mobility test progression revealed that two and eight weeks after surgery, the TMT and TMT + SC-treated animals were able to walk for longer distances than mice that received DMEM only (Figure 4A–H). Quantification showed that TMT + SC animals achieved the highest velocity compared to other groups (Figure 4I).

### Regenerating axons formed neuromuscular junctions

**Figure 5:**
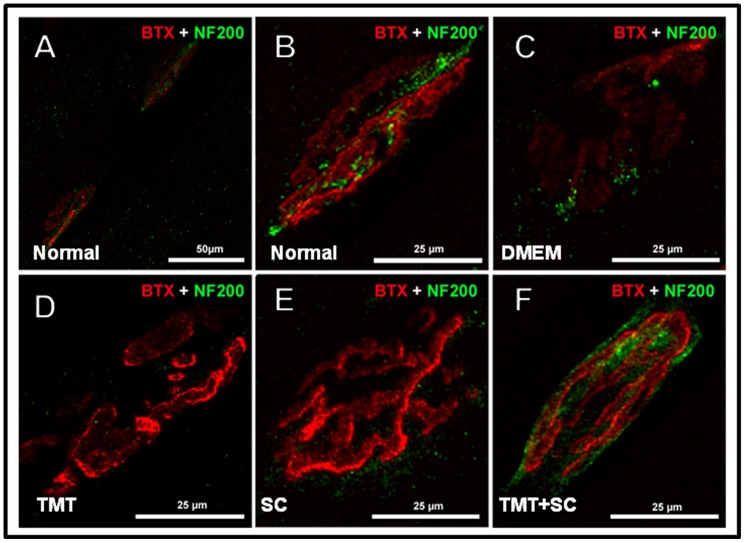
Motoneurons regenerate and form neuromuscular junctions. Longitudinal sections of gastrocnemius muscle showing end plates identified with α-bungarotoxin binding to acetylcholine receptors (red) and nerve terminal stained with neurofilament-200 (green). (A and B) Normal animal used as positive control; most end plates exhibited a “pretzel-like” shape with precise apposition of motor nerve terminals to the motor endplates, indicating that the endplate is perfectly innervated. (C, D and E) DMEM, TMT and SC groups showed neuromuscular junctions with disorganized and enlarged morphology, and exhibited poor and fragmented NF-200 staining. (F) The TMT + SC group displayed more “pretzel-like” junctions, similar to normal animal. Bar: (A) 50 µm (B, C, D, E and F) 25 µm.

The functional and morphological results strongly suggested that regenerated nerves were able to reinnervate target muscles of treated animals. To confirm this hypothesis the neuromuscular junctions were qualitatively analyzed in each group. In normal animals, the staining of junctions by anti-neurofilament and α-Bungarotoxin has a “pretzel-like” shape, with precise apposition of motor-nerve terminals to the motor endplates (Figure 5A and 5B). We were able to visualize the neuromuscular junctions in all surgically transected groups, suggesting that the regenerated axons were indeed able to reinnervate target muscles (Figure 5). However, in the DMEM (Figure 5C), TMT (Figure 5D) and SC groups (Figure 5E), most of the junctions were enlarged and disorganized, with weak labeling for neurofilaments (Figure 5F). In contrast, the TMT + SC group showed a much better apposition of motor-nerve terminals to the endplate, with neuromuscular junctions closely resembling those of the normal animal.

### Polycaprolactone tubes are a suitable substrate for the regenerating nerve and progressively degrade *in vivo*

**Figure 6:**
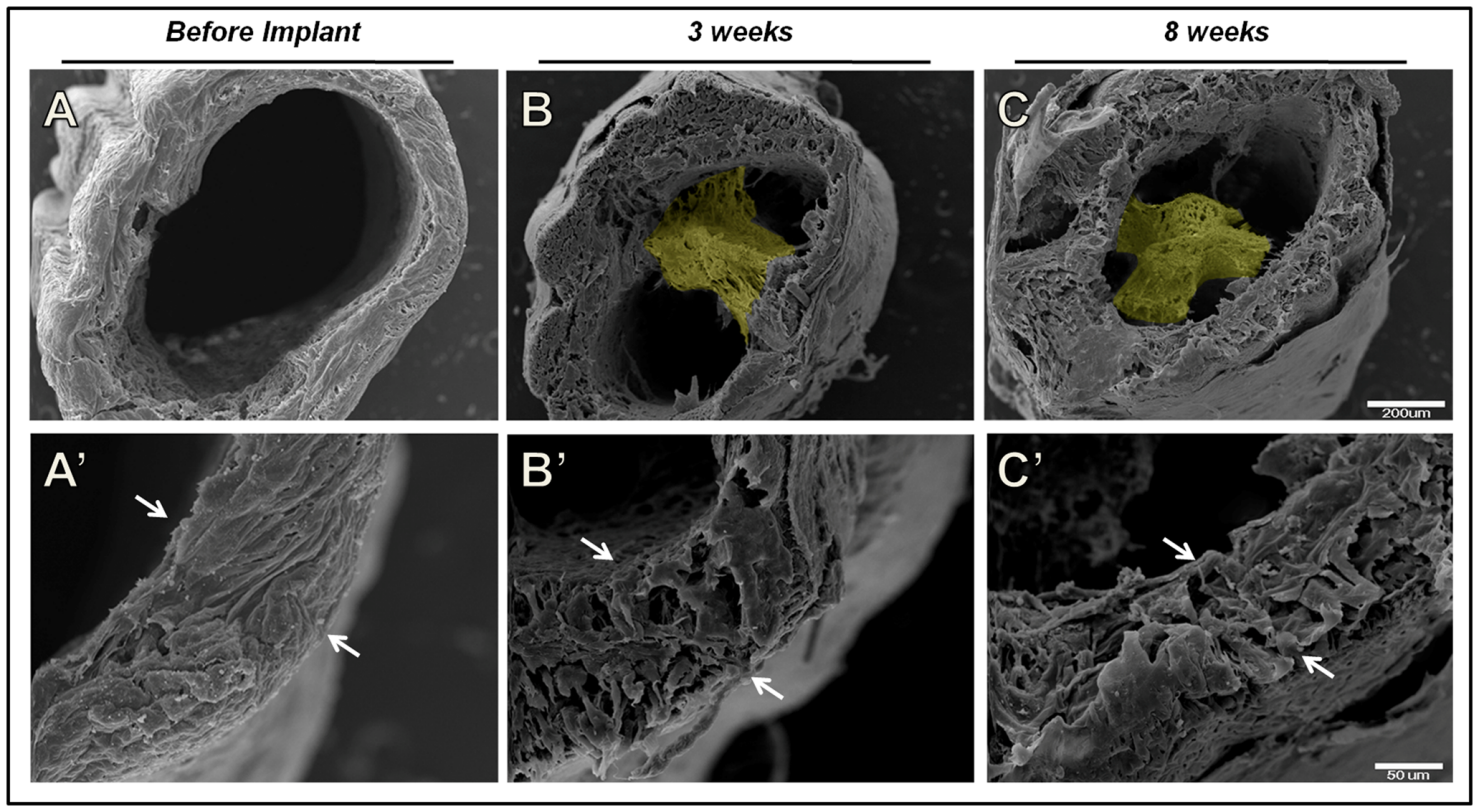
Scanning electron microscopy of polycaprolactone tubes. (A and A′) Appearance of the empty tube before being implanted (A), and higher magnification of the tube wall showing its normal aspect (A′); (B and B′) tube containing the growing nerve in its interior (B) and the tube wall starting to disintegrate three weeks after the implant (B′); (C and C′) regenerated nerve within the tube (C′) and tube wall in process of disintegration (C′) eight weeks after implantation. Arrows delimit the thickness of the tube wall, which is clearly reduced over time. Bar: 200 µm (A, B and C) and 50 µm (A′, B′ and C′).

We observed the ultrastructural features of the polycaprolactone conduit prior to implantation (Figure 6A and 6A′), 3 and 8 weeks after being implanted (Figure 6B and 6B′; 6C and 6C′, respectively), by scanning electron microscopy. Cross sections of tube showed the growing nerve inside and in close contact with the tube wall (Figure 6B and C). In the course of time, the tube wall became thinner, showing its biodegradable property (Figure 6A′, B′ and C′).

### Transplanted Schwann cells functionally reintegrate into the tissue

**Figure 7:**
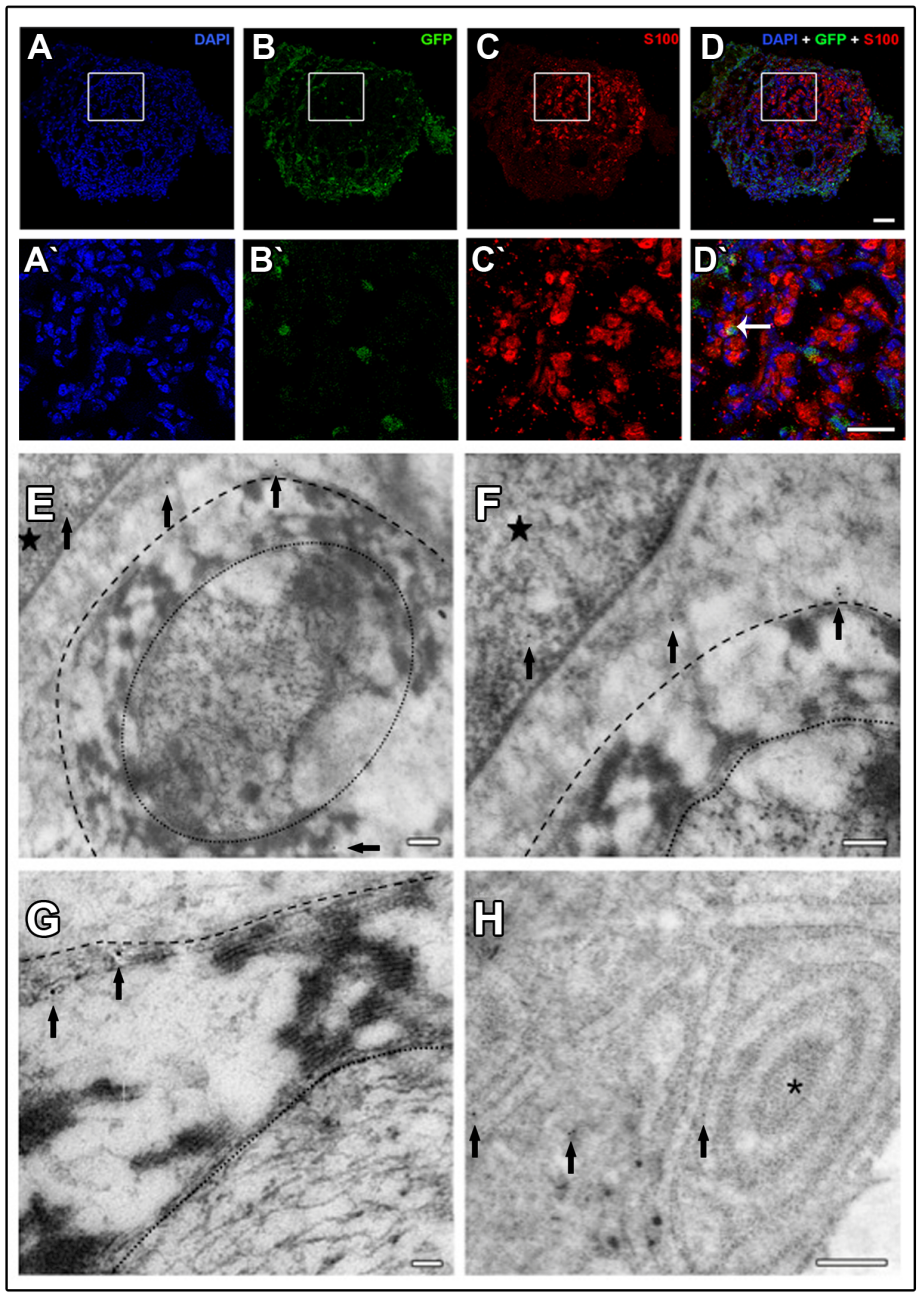
Tracking Schwann cells eight weeks after injection. (A and A′) cell nuclei labeled with DAPI; (B and B′) GFP^+^ Schwann cells; (C and C′) SC stained for S-100; (D and D′) Overlay images. Arrow indicates transplanted Schwann cell expressing GFP and S-100. (E–H) Immunoelectronmicroscopy for GFP^+^ Schwann cells. (E) Schwann cell labeled with colloidal gold (arrows) myelinating a regenerated fiber; (F) Higher magnification of (E) showing gold particles in SC nucleus and cytoplasm. (G) Tagging with colloidal gold in the myelin sheath of a regenerated myelinated fiber; (H) Tagging with colloidal gold showing well-developed RER in a Schwann cell. (Arrows) Colloidal gold particles; (Stars) Schwann cell nuclei; (Dashed line) External limit of a nerve fiber myelinated by GFP^+^ Schwann cell; (Dotted line) Axon limit; (Asterisk) Schwann cell RER. Bar: 20 µm (A, A′, B, B′, C, C′, D and D′); Bar: 0.2 µm (E and F); 100ηm (G); 0.5 µm (H).

The survival and presence of green fluorescent protein in GFP^+^ Schwann cells inside the tube were evaluated 8 weeks after transplantation. In the distal segment of the regenerated sciatic nerve, GFP^+^ cells were readily visible (Figure 7A, 7A′, 7B and 7B′) and also expressed S-100 (Figure 7C, 7C′, 7D and 7D′). Immunogold images revealed that GFP^+^ cells were in direct contact with myelinated fibers (Figure 7E–H). Colloidal gold particles were easily observed in both SC cytoplasm and nucleus, as well as in the myelin sheath of myelinated fibers (Figure 7E, 7F and 7G). We also detected labeled Schwann cells exhibiting a prominent rough endoplasmic reticulum (RER) in the cytoplasm (Figure 7H).

### Combined therapies increase levels of trophic factors *in vivo*

**Figure 8:**
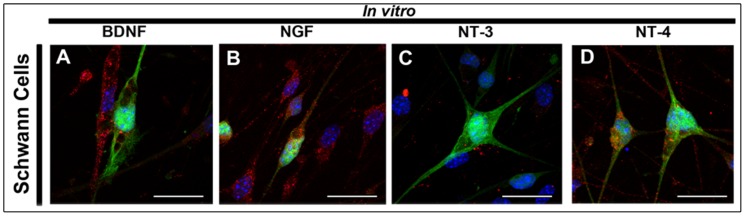
Analysis of neurotrophic factor expression by Schwann cells *in vitro*. Schwann cells were able to express BDNF, NGF, NT-3 and NT4 in culture (A, B, C and D, in Red); (Blue) Nuclei stained with DAPI; (Green) Expression of green fluorescent protein (GFP) by Schwann cells. Bar: 25 µm (A–D).

**Figure 9:**
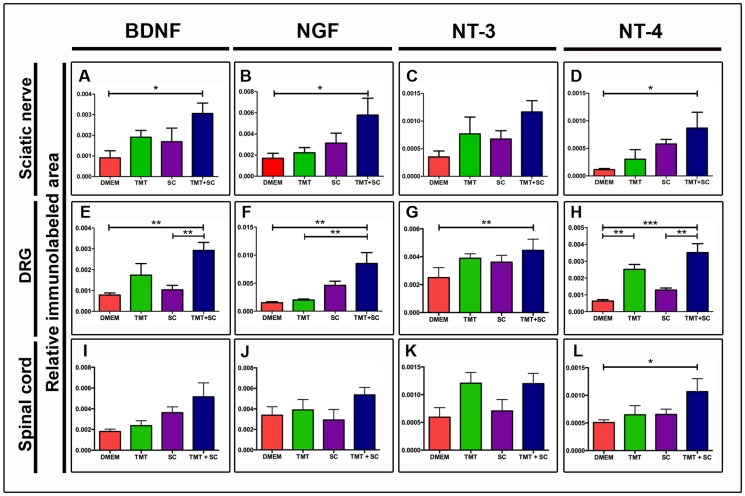
Quantification of relative immunoreactive area stained with BDNF, NGF, NT3 and NT4 in the sciatic nerve, dorsal root ganglion and spinal cord (A–L). Analysis of BDNF, NGF and NT4 expression on sciatic nerve showed significant values when the TMT + SC group was compared to the DMEM group (A–D). The DRG quantification on the TMT + SC group presented significantly higher levels of all analyzed trophic factors in relation to the DMEM group (E–H). Trophic factor staining in spinal cord presented a significant difference in the NT4 fluorescence intensity between TMT + SC and DMEM groups (I–L). Values represent mean ± SEM, *P<0.05, **P<0.01, ***P<0.001.

Analysis of *in vitro* expression showed that Schwann cells were positively stained for BDNF (Figure 8A), NGF (Figure 8B), NT-3 (Figure 8C) and NT-4 (Figure 8D). Eight weeks after cell transplantation, sciatic-nerve, DRG and spinal cord sections from transected animals showed immunoreactivity for all trophic factors (Figure 9A–L). Quantification of trophic factors immunoreactive area, relative to total nerve area, showed a significant difference between TMT + SC and DMEM groups for BDNF (Figure 9A; 0.003059±0.0005070 and 0.0009073±0.0003455, respectively, *P*<0.05), for NGF (Figure 9B; 0.005780±0.001606 and 0.001699±0.0004659, respectively, *P*<0.05) and for NT-4 (Figure 9D; 0.0008682±0.0002859 and 0.0001162±0.00001860, respectively, *P*<0.01). Immunoreactivity for NT-3 showed no significant difference between groups (Figure 9C).

The quantification of all analyzed trophic factors in the dorsal root ganglia of the TMT + SC group revealed significantly higher levels in comparison to the DMEM group. TMT + SC group (0.002918±0.0003860) had a larger immunopositive area for BDNF compared to DMEM (0.000782±0.0002193) and SC (0.001031±0.0002140, *P*<0.01) groups (Figure 9E). Quantification of NGF labeling presented significant difference between TMT + SC group (0.008512±0.001935) compared to DMEM and TMT groups (0.001512±0.0001766 and 0.001989±0.0001795, *P*<0.01, respectively; Figure 9F). Analysis of NT3 in the TMT + SC group (0.004458±0.0004005) showed significant difference only compared to DMEM group (0.002504±0.0003557, *P*<0.01; Figure 9G). Finally, levels of NT4 staining in the TMT + SC group (0.003511±0.0005275) were significantly higher than in DMEM and SC groups (0.0006326±0.0001749 and 0.001287±0.0001189, *P*<0.001 and *P*<0.01, respectively). NT4 levels were also different between TMT and DMEM groups (0.002513±0.0002927 and 0.0006326±0.0001749, *P*<0.01, respectively; Figure 9H).

Staining of trophic factors in the spinal cord showed a significant difference in the intensity of fluorescence between TMT + SC and DMEM groups only for NT4 (0.001067±0.0002359 and 0.0005077±0.0001098, *P*<0.05, respectively; Figure 9L). Immunoreactivity for the other trophic factors was similar among groups (Figure 9I–K).

### Therapies influence neuronal survival

**Figure 10:**
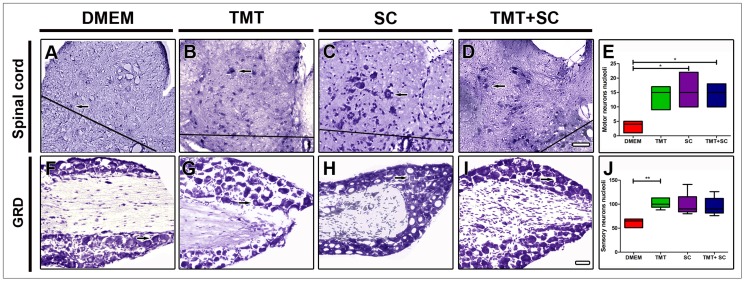
Quantification of neurons nucleoli in spinal cord and in dorsal root ganglion (D). Cross sections through the spinal cord (A–D) and longitudinal sections through DRG (F–I), 8 weeks after sciatic nerve transection. Arrows show examples of quantified nucleoli. (E) and (J) represent quantification of motor neurons in the ventral horn spinal cord and sensory neurons in DRG. Values shown are mean ± SE; *P<0.05. Scale bars: 50 µm.

To evaluate the efficiency of combined therapies on the survival of dorsal root ganglion and spinal cord neurons, the L4 lumbar segment and L4 dorsal root ganglions were analyzed eight weeks after nerve injury (Figure 10A–J). The total number of motor neurons in L4 segments of the DMEM group (Figure 10A–D) was significantly lower (3.2±0.91) than the ones found on the SC and TMT + SC groups (15.80±2.69 and 14.20±1.80, respectively; Figure 10E), indicating that SC and TMT + SC treatments protected motor neurons from cell death. Counts of DRG sensory neurons (Figure 10F–I) revealed that the TMT group (102.6±4.73) had significantly more neurons than the DMEM group (61.0±4.14). The SC and TMT + SC (97.80±10.96 and 95.40±8.424, respectively) groups had a slight, but not significantly larger number of motor neurons compared to DMEM group (Figure 10J). These results suggest that TMT was more effective in protecting sensory neurons than SC treatment or the combination of both. Table 1 summarizes the results.

**Table 1:** Summary of the results.

	*Normal*	*DMEM*	*TMT*	*SC*	*TMT + SC*
***Number of myelinated fibers***	2840±55.01	1160±293.30	1877±211.30	1793±250.6	2187±111.9*
***Myelin area***	9.369±0.14	4.628±0.78	5.503±0.73	7.601±0.74	8.195±1.09*
***Fiber area***	17.125±0.11	10.64±0.12	13.605±0.50	14.494±1.15	13.248±1.25
***Axon area***	7.778±0.06	5.374±0.91	7.169±0.63	6.208±1.13	6.193±0.71
***Number of blood vessels***	367.7±14.68	243±14.33	406.7±13.53**	392. 4±28.90*	367.7±14.68**
***Sciatic function index***	---	91.59±4.14	71.42±1.40	73.70±3.10	64.55±2.32*** ^+^
***Global mobility test***	---	5.771±0.08	8.279±0.29	6.632±0.16	9.516±0.61
***Motor neurons nucleoli***	---	3.20±0.92	13.4±1.83	15.8±2.69*	14.2±1.80*
***Sensory neurons nucleoli***	---	61.0±4.15	102.6±4.74**	97.8±10.96	95.4±8.42

**Values represent mean ± SEM,**

* ***P*** **<0.05,**

** ***P*** **<0.01,**

*** ***P*** **<0.001.**

Comparison showing statistically significant differences between the treated groups and DMEM (represented by *); between the TMT + SC and SC groups (represented by +).

## Discussion

The peripheral nervous system has a well-documented ability to regenerate, and yet functional recovery in severe traumas such as complete nerve transection is still clinically unsatisfactory and often results in disability and decreased quality of life for affected individuals [35], [36]. Thus, in order to optimize the regenerative process and reach full functional recovery, development of new therapeutic approaches and the combination of different existing strategies are necessary.

In the present study, we combined multiple repair strategies, namely: surgical repair with a tubular conduit; grafts of cultured Schwann cells; and treadmill training. This combination accelerated the regenerative process of the sciatic nerve, increasing the number of myelinated fibers and myelin area, as well as improving recovery of motor function. We also observed a better structural organization of motor-nerve terminals, which suggested a more efficient reinnervation of the gastrocnemius muscle.

There is a general agreement that functional recovery is improved when the time for reinnervation is short [37], mainly because the regenerative potential of neuron cell bodies and the plasticity of Schwann cells decline over time. Therefore when the distance between the injury site and the target organ is too long, a reduced number of motor axons can regenerate and make functional connections with denervated muscle fibers [38]. Hence, the growth rate and the number of axons may have direct implications for functional recovery. The g-ratio is an index that reflects the axonal myelination that leads to an appropriate conduction of action potentials. The optimal g-ratio rate for myelinated fibers from sciatic nerve was defined as 0.55–0.68 [39]. In our experiment, the animals treated with combined therapies achieved a larger number of myelinated fibers, a larger myelin area and increased g-ratio values. Together, these results may suggest an improved reinnervation of the target organ.

Following sciatic nerve injury and repair, the analysis of walking tracks, quantified by the sciatic function index has proven to be a reliable method for evaluating functional recovery. It provides an easily obtained, serial and direct assessment of function. Previous studies by our group have already shown a clear correlation between morphological and functional recovery [5], [31]. Our work showed that TMT + SC-treated animals exhibited an improved functional recovery compared to other groups possibly due to the better morphological results.

The ultimate indication of muscle reinnervation is whether the nerve is connected to the myofiber. We therefore analyzed qualitatively the morphology of neuromuscular junctions. In the TMT + SC-treated group we saw an improved structural organization of junctions which was similar to a normal animal. We also observed a better apposition of motor nerves terminals in relation to the endplate. Most junctions found in DMEM, TMT and SC-treated groups were expanded and disorganized, with deficient staining against NF-200. This deficiency can disrupt the addressing of presynaptic vesicles toward the endplate. Despite the fact that we have not performed quantification of the data, this analysis supports the functional and morphological improvement found in the TMT + SC-treated group.

Tubular guides serve as a bridge between the distal and proximal segments of the nerve, and their use has been considered a promising alternative to autologous nerve graft [40]. Two types of biodegradable tubular prostheses, collagen and polycaprolactone tubes, were used here. Both were previously tested by our and other groups, and are a suitable substrate for adhesion and elongation of the growing nerve fibers [6], [24], [31], [41]. Ultrastructural analysis of the tubes eight weeks post-surgery, revealed clear signs of an initial degradation process. This is consistent with previous reports, and indicates an adequate degradation rate [32].

In addition to the tubular conduit, cell therapy with Schwann cells was used to improve regeneration. These cells are the principal support cells in peripheral nervous system, and when used in cell therapy, possess the remarkable ability to promote nerve regeneration in both central and peripheral nervous systems [42], [43]. This therapeutic value is mainly attributed to their potential to produce trophic factors, which are important for the regenerative process in the peripheral nerves [44]. An important issue when using cell therapy is the viability of the transplanted cells in the host tissue. Our results demonstrated the presence of Schwann cells in the middle segment of the regenerated nerves up to 56 days after injury. These findings are consistent with previous reports, which identified Schwann cells in the host tissue for up to 16 weeks after they were injected into polycaprolactone or collagen tubes [10], [45]. Furthermore, to the best of our knowledge, transplanted Schwann cells have never been shown to actually functionally incorporate into the tissue. Through an ultrastructural approach, we have demonstrated for the first time that transplanted Schwann cells can form a myelin sheath around regenerating axons; also, these cells exhibited a prominent rough endoplasmic reticulum, which indicates that the transplanted cells were active and probably secreting proteins. The capability of transplanted cells to fully reintegrate with the nerve represents an advantage in terms of cell-based therapies, as it suggests that exogenous Schwann cells can be recognized as native to the tissue.

Treadmill training has been advocated by researchers and physiotherapists for its benefits to treat spinal cord injuries [15], [46]. Recently, attention has also turned to the potential of physical activity in the treatment of peripheral nerve injuries. Some experimental studies have shown that treadmill exercise, even in very small doses, can enhance axon regeneration and speed functional recovery after sciatic nerve compression in mice and in rat models [18], [19], [27], [47]. However, this is one of the first studies to provide direct evidence of the positive effects of the combination of cellular therapy seeded in a tubular conduit, with treadmill training, in peripheral-nerve regeneration after transection injury in mice.

Several elements are essential for proper nerve regeneration, and neurotrophic factors are particularly important [48], since they can regulate neuronal survival, promote axonal and neurite regeneration, and reinnervation of target organs [38], [49]–[52]. Schwann cells are described to exert neurotropic and guiding effects for axonal projection by secreting neurotrophins and adhesion molecules [44]. The effects of exercise found in this study may also be attributable to the upregulation of neurotrophins, since exercise is known to enhance the levels of BNDF in regenerating neurons [20]. We observed that Schwann cell and treadmill training treatments increased the expression of neurotrophins in the sciatic nerve (BDNF, NGF and NT-4), DRG (BDNF, NGF, NT3 and NT4) and spinal cord (NT4). This effect might be attributed to direct secretion of trophic factors by the injected cells and/or to release promoted by the treadmill training [53], [54]. Taken together, these evidences may explain the greater degree of fiber myelination observed in the TMT + SC group.

Given that one of the roles of neurotrophins is to regulate neuronal survival [55], we also analyzed the number of dorsal root ganglion and spinal cord neurons. Our untreated group (DMEM) showed a substantial reduction in both DRG and spinal cord neuronal survival. Meanwhile, all the employed treatments, albeit not always significant, exerted some degree of protection to sensory and motor neurons.

The translation of pre-clinical studies to an alternative therapeutic approach combining therapies to treat PNS injuries in humans is necessary, but requires further investigation. Developing a protocol to expand human Schwann cells from a small biopsy sample to a large number of cells is still a challenge, and the ability of cultured human Schwann cells to produce myelin in the tissue requires further demonstration. Moreover, it is also important to establish an individual treadmill-training protocol appropriate for each type of injury.

In summary, this study provides evidence that combined therapies improve sciatic-nerve regeneration after a traumatic nerve injury in mice. These strategies are promising in aiding regeneration after nerve damage, and might benefit patients with neurodegenerative diseases in the future.
